# Cytotoxicity and dentin composition alterations promoted by different chemomechanical caries removal agents: A preliminary *in vitro* study

**DOI:** 10.4317/jced.58208

**Published:** 2021-08-01

**Authors:** Fernanda-Rodrigues Guedes, Jéssica-Fernanda-Sena Bonvicini, Gabriela-Leite de Souza, Washington-Henrique-Themoteo da Silva, Camilla-Christian-Gomes Moura, Luiz-Renato Paranhos, Ana-Paula Turrioni

**Affiliations:** 1 Department of Pediatric Dentistry, School of Dentistry, Federal University of Uberlandia, Uberlandia, MG, Brazil; 2 Department of Endodontics, School of Dentistry, Federal University of Uberlandia, Uberlandia, MG, Brazil; 3 Department of Community and Preventive Dentistry, School of Dentistry, Federal University of Uberlandia, Uberlandia, MG, Brazil

## Abstract

**Background:**

The use of chemomechanical agents for caries removal has been indicated as a non-invasive treatment option; however, their possible deleterious effects on the dental-pulp complex have been insufficiently studied. This study assessed the direct cytotoxicity of two chemomechanical caries removal agents (Brix 3000™ - BX and Papacarie Duo™ - PD) on pulp cells from deciduous teeth, as well as to assess the morphology and chemical compositions of the dentin surface after the application of these materials.

**Material and Methods:**

The cells were seeded (50,000 cells/cm²) in a culture medium (DMEM with 10% fetal bovine serum - FBS). After 24 hours, the BX and PD materials were added to 1:20, 1:100, and 1:1000 dilutions. Non-exposed cells were considered as the control group. The viability test (MTT), Trypan Blue assay (TB), and cell morphology (Scanning Electron Microscopy - SEM) were performed after 24 hours of agent application. For the SEM and chemical (energy-dispersive X-ray spectrometry - EDS) dentin evaluation, 0.3-mm-thick dentin discs were obtained and divided into control group (no treatment) and surfaces covered with 37% phosphoric acid, BX, or PD. Data were compared by one-way ANOVA and Tukey’s test (*p*<0.05).

**Results:**

Decreases in cell viability and numbers of viable cells were observed for both materials, at all dilutions, when compared with the control group (*p*<0.05). The BX and PD materials did not cause visually perceptible changes, according to SEM, on the surfaces of dentin discs. The EDS analysis did not indicate a statistically significant difference in the levels of calcium (Ca) and phosphorus (P) between the materials and the control group (*p*>0.05).

**Conclusions:**

Both materials showed cytotoxicity when in direct contact with the pulp cells from deciduous teeth, and the BX material presented lower cytotoxicity than the PD material. Moreover, both materials did not significantly change the dentin composition.

** Key words:**Cell culture, cytotoxicity, dental pulp, papacarie, primary teeth.

## Introduction

Caries is a multifactorial disease caused by an imbalance in the demineralization and remineralization processes of hard dental tissues, causing progressive destruction (1). Regarding the formation of the caries lesion, dentin is classified into two layers, differentiated macroscopically by the properties of resistance to cutting and staining (2). The most superficial dentin, called ‘infected’, has a loss of integrity due to collagen degeneration caused by microorganisms, resulting in a more soft and moist tissue. This layer must be removed, leaving only the affected dentin, which has more rigid characteristics and is capable of remineralization(3). In clinical practice, it is difficult to distinguish between these layers, since there is no precise technique for recognizing the exact limit between them (4). However, it is known that obtaining good tissue repair and decreasing the risk of exposure require that the dentist maintain the affected dentin (5), justifying the need to standardize the best technique for carious tissue removal.

Removing caries by the conventional method usually causes discomfort, anxiety, and pain to the patient because it requires high-rotation burs and does not remove carious and healthy tissues selectively, i.e., it removes both infected and affected dentin. In most cases, this procedure requires local anesthesia, which also decreases patient acceptance (6). Dentistry is constantly searching for treatments that cause less discomfort to patients. This is also important for dentists working in the context of the pandemic, which requires professional preventive and minimally invasive approaches to the management of caries (7).New technologies such as cavity preparation by oscillation (ultrasound) (8), lasers (9), ozone (10), and chemomechanical caries removal agents (11) have been used. Most of these methods involve a high cost and may lead to tooth hypersensitivity, contributing to painful sensations, especially in children.Chemomechanical caries removal is a non-invasive technique that aims to dissolve necrotic tissues, facilitating the removal of the tissue softened manually with blunt-tip instruments. This method has been reported as less painful and more tolerable when compared with the traditional method (12).

The chemomechanical caries removal agents, initially based on n-monochloroglycine and sodium hypochlorite, appeared in 1972, but they removed the carious tissue slowly (13). In the 1990s, Carisolv™ was introduced, consisting of a gel with two components: one based on 0.5% sodium hypochlorite and the other based on amino acids (glutamic acid, leucine, and lysine), sodium chloride, erythrosine, and distilled water. Although it was considered effective and easy to handle, the product was expensive and required customized tools (14). In 2003, due to a need to promote the use of the chemomechanical removal method in the Brazilian public health system, a gel (Papacarie™) was developed, the main component of which was papain - an enzyme similar to human pepsin, extracted from the papaya peel. This enzyme breaks the denatured collagen fibers, allowing for easy removal with handpieces (15). The agent is also composed of chloramine, which chemically softens the carious dentin and connects to the degraded collagen portion and toluidine blue, with antimicrobial action. Its use presented satisfactory results when compared with atraumatic restorative treatment and other chemomechanical removal agents, in both permanentand deciduous teeth (16). More recently, a new papain-based agent (Brix 3000™) was introduced to the market in 2017, with major differences in composition. It presents a higher papain concentration and has been suggested to have anti-inflammatory properties, which may favor the recovery of pulp tissue (17).

Besides the different options for the chemomechanical removal of carious tissue, the information regarding pulp cytotoxicity from the use of this type of technique is scarce, especially when the materials are applied in deep cavities presenting risk of pulp exposure. This study aimed to assess the cytotoxicity of two chemomechanical caries removal agents (Brix 3000™ and Papacarie Duo™) when applied in different dilutions directly to the pulp cells of deciduous teeth, as well as to assess the morphology and chemical composition of the dentin surfaces after the application of these materials. The null hypotheses were that: 1ªthe materials tested would not present cytotoxicity for the pulp cells of human deciduous teeth when placed directly on the cells in diluted form; and 2ªthe materials tested would not change the elementary structure and chemical composition of the dentin surface after *in vitro* smear layer formation.

## Material and Methods

The CRIS (Checklist for Reporting *in vitro* Studies) tool (18)was used for designing and writing the results according to the recommendations for *in vitro* studies. Moreover, the entire method of the present *in vitro* study was performed according to the International Organization for Standardization (ISO) guidelines 10993-5: 2009.

-Obtaining pulp cells from deciduous teeth (PCDD)

Pulp cells from deciduous teeth (PCDD) were obtained from three healthy teeth collected at the School of Dentistry of the Federal University of Uberlândia (UFU), Brazil. The pulp tissue was immersed for 1 h at 37°C in tissue digestion solution (3 mg/mL of collagenase type I, Sigma-Aldrich, St. Louis, MO, USA, and 4 mg/mL of dispase, Sigma-Aldrich). The cells obtained were seeded in 25-cm² flasks and incubated for four days at 37°C, with 5% CO2 (19).

-Experimental protocol for cell culture

The experimental protocol was performed with cells from the 4th to 6th passages. The pulp cells were seeded (20,000) in 24-well plates (Costar Corp., Cambridge, MA, USA) with DMEM (Sigma-Aldrich) supplemented at 10% with bovine fetal serum (Gibco, Grand Island, NY, USA), with 100 UI/mL of penicillin, 100 μg/mL of streptomycin, and 2 mmol/L of glutamine (Gibco). They were maintained in an incubator with 5% CO2 at 37°C, and after 24 h, the materials were added. Two gels were used for the cytotoxicity analysis: Papacarie Duo™- PD (Fórmula e Ação F&A, Laboratório Farmacêutico Ltda, São Paulo, SP, Brazil) and Brix 3000™ - BX (BRIX S.R.L., Província de Santa Fé, Argentina).

The experimental and control groups were distributed as follows for the metabolism analysis and the number of viable cells (n=8): G1- Control (DMEM), G2 –BX 1:20, G3 – BX 1:100, G4 – BX 1:1000, G5 – PD 1:20, G6 – PD 1:100, and G7 – PD 1:1000. The materials were diluted in DMEM, according to each concentration. The samples were homogenized with a pipette and standardized. To justify the dilutions used in the present study, a previous pilot study developed by the research group showed that the IC50 (half of the maximum inhibitory concentration) of the BX material was the 1:20 dilution (data not presented). From this dilution, two higher ones were selected (1:100 and 1:1000) for comparison between groups. The tests were performed 24 h after the contact with the materials (20).

-Cell viability (MTT assay)

Cell viability was assessed with the methyl-tetrazolium assay (MTT). This analysis allowed for determination of the activity of the succinic dehydrogenase enzyme connected to the internal mitochondrial membrane, which intervenes in cell respiration and may be considered the cell metabolic rate. Each well of the culture medium received 900 µL of DMEM without bovine fetal serum and 100 µL of MTT solution (5 mg/mL in PBS; Sigma-Aldrich). After four h in the incubator at 37°C, the solution was replaced with 700 µL of acidified isopropanol (0.04 N of HCL), to dissolve the formazan crystals resulting from the methyl-tetrazolium salt cleavage by the succinic dehydrogenase enzyme in the viable cells, producing a homogeneous bluish solution. Three parts of 100 µL of each sample were transferred to a 96-well plate (Costar Corp.) and analyzed in a spectrophotometer (Thermo Plate, Shenzhen, China) with a 570-nm filter.

-Viable cell count (Trypan Blue assay)

The Trypan Blue assay (TB) was used to assess the number of viable cells after the chemical removers were applied. This test assesses the total number of viable cells in the samples directly, because the TB dye can penetrate only the porous and permeable membranes of damaged dead cells, which is detecTable by microscopy. The culture medium was removed and the cells were trypsinized with 300 µL of 0.25% Trypsin (Invitrogen, Carlsbad, CA, USA) for 10 min.After this time, 50 µL of the cell suspension and 50 µL of 0.04% Trypan Blue solution (Sigma-Aldrich) were transferred to a 96-compartment plate and incubated for 2 min at room temperature. Ten microliters of each sample were transferred to a hemocytometer that, aided by a manual counter, allowed for counting of the total numbers of viable and non-viable cells with an inverted light microscope (Nikon Eclipse TS 100, Nikon Corporation, Tokyo, Japan). Non-viable cells presented blue-stained cytoplasm due to the penetration of the TB solution within the cells that presented a rupture of the plasma membrane. The number of viable cells was determined by subtracting the number of non-viable cells from the total number of cells.

-Morphological analysis by Scanning Electron Microscopy (SEM)

A qualitative analysis of the cell culture was performed for the assessment of cell morphology after the contact with the chemical removers, complementing the cell viability analysis.The cells were seeded in coverslips and fixed for 1 h in 2.5% glutaraldehyde (Sigma-Aldrich). After fixation, each well was washed 3x with 1 mL of PBS (5 min per wash), and the cells were post-fixed for 60 min in 200 μL of 1% osmium tetroxide (Sigma-Aldrich). The samples were dehydrated in ascending exchanges of ethanol (30%, 50%, and 70%, 2x 95% and 2x 100% - 30 min in each solution), then dried with the 1,1,1,3,3,3-hexamethyldisilazane solvent (HMDS - ACROS Organics, Rutherford, NJ, USA) and maintained in a desiccator for one week. The samples were fixed in stubs, metalized with gold, and analyzed in a scanning electron microscope (SEM, JEOL-JMS-T33A Scanning Microscope, JEOL Inc., Peabody, MA, USA).

-Analysis of morphology and elementary chemical composition of dentinal discs by energy-dispersive X-ray spectrometry (EDS)

From 8 human permanent teeth (third molars), eight 0.3-mm-thick dentin discs were obtained by means of a diamond disc (11-4254, 4”x 0.012”/15LC series, Diamond Wafering blade, Buehler Ltd., Lake Bluff, IL, USA) coupled to the appliance for serial cutting (ISOMET 1000, Buehler) for the chemical composition analysis.The discs were removed close to the pulp, and the thickness of 0.3mm was used to simulate a greater challenge of the pulp tissue, an extremely deep cavity. The dentin disc surfaces were sanded and leveled with 400- and 600-granulation sandpapers (T469-SF-Noton, Saint-Gobam Abrasivos Ltda., Jundiai, SP, Brazil), and during this procedure, the discs were often assessed with the help of a digital caliper (Model 500-144B, Mitutoyo Sul America Ltda. SP, Brazil) to ensure that the final thickness would reach 0.3 mm. The discs were divided into four groups and placed on a 24-well plate (Costar Corp.) for the direct application of the product. Aided by an insulin syringe, a 270-mg quantity of the material was applied to the occlusal surfaces of the dentin discs until they were filled homogeneously. In group 1, there was no agent application (control); in group 2, 37% phosphoric acid was applied (Condac 37, FGM, Joinville – SC, Brazil) for 15 s; in group 3, Papacarie Duo™ was applied for 30 s; and in group 4, Brix 3000™ was applied for 2 min. These times are the minimum recommended by the manufacturers. After the applications, the discs were washed 3x with distilled water and subjected to the SEM analysis protocol described previously, except for the fixation phase in 1% osmium tetroxide.

Besides the images produced, there was a chemical analysis produced by SEM withan EDS detector (energy-dispersive X-ray detector, Oxford, 51-ADD0048, Cambridge, England), which separates the characteristic X-rays from the different elements in an energy spectrum and allows for elementary microanalyses of the sample. The elements considered for the statistical analysis were calcium and phosphorus. For each dentin disc, four different points of the dentin surface were assessed, resulting in a total n of eight per group. The points were standardized, including the central and peripheral regions of the dentinal tissue.

-Statistical analysis

The data were tabulated in spreadsheets and subjected to analysis by SPSS version 18.0 statistical software. Respecting the distribution of data, one-way ANOVA complemented by Tukey’s test (5% significance) was used for cell cytotoxicity analysis and EDS, and the cytotoxicity data were transformed into a percentage, with the control group considered as 100%.

## Results

-Cell viability by the methyl-tetrazolium assay – MTT

The results showed that all the dilutions of the different materials differed from the DMEM control group (*p*<0.05). It was observed that the concentration increase resulted in a viability decrease, showing the highest cytotoxicity in the different materials at the 1:20 dilution. However, the viability of cells exposed to the Papacarie Duo material diluted at 1:20 was considerably lower when compared with that of the Brix 3000 group at the same dilution, with values of 19.8% and 52.5%, respectively (*p*<0.05). The material that came closer to the control was Brix 3000, and the highest viability was obtained at the 1:1000 dilution (81.1%); there was not a considerable value range. At the highest concentration of the material, viability was 52.5%. In contrast, the Papacarie Duo group showed a higher variation, and the 1:1000 and 1:20 dilutions presented values of 74.7% and 19.8%, respectively (Table 1).

**Table 1:**
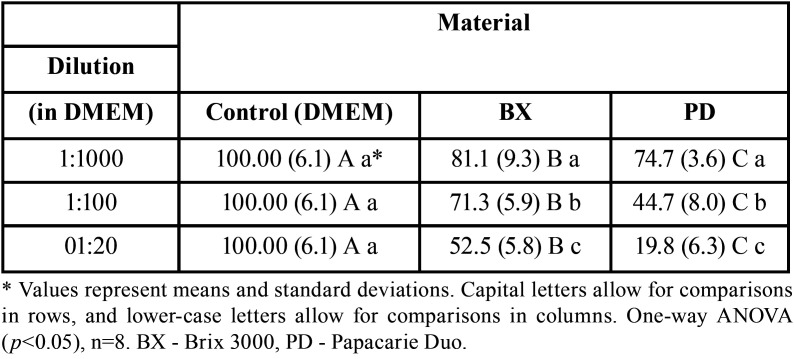
Cell viability (MTT) presented by the pulp cells of deciduous teeth, considering the different dilutions and materials used.

-Numbers of viable cells by the Trypan Blue assay

As for the Trypan Blue results, the results were similar to those for MTT. Both tests showed that the higher the concentration, the lower the number of viable cells. Moreover, the Brix 3000 material also presented fewer discrepancies relative to the control group. The 1:1000 dilution of the Brix 3000 material did not show a statistically significant difference from the control group (*p*>0.05). The group with the highest concentration of the Papacarie Duo material (1:20) presented the lowest number of viable cells (35.9%), following the pattern of results found in the MTT assay. For each material, there was no statistically significant difference between the 1:1000 and 1:100 dilutions (*p*>0.05). The difference occurred only when the materials with the lowest and highest concentrations were compared (1:1000 and 1:20, *p*<0.05) (Table 2).

**Table 2:**
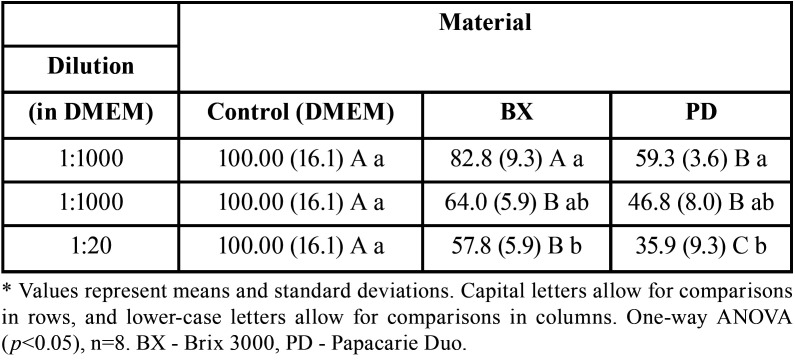
Numbers of viable cells by the Trypan Blue assay presented by the pulp cells of deciduous teeth, considering the different dilutions and materials used.

-Morphology of pulp cells by Scanning Electron Microscopy (SEM)

**Figure 1:**
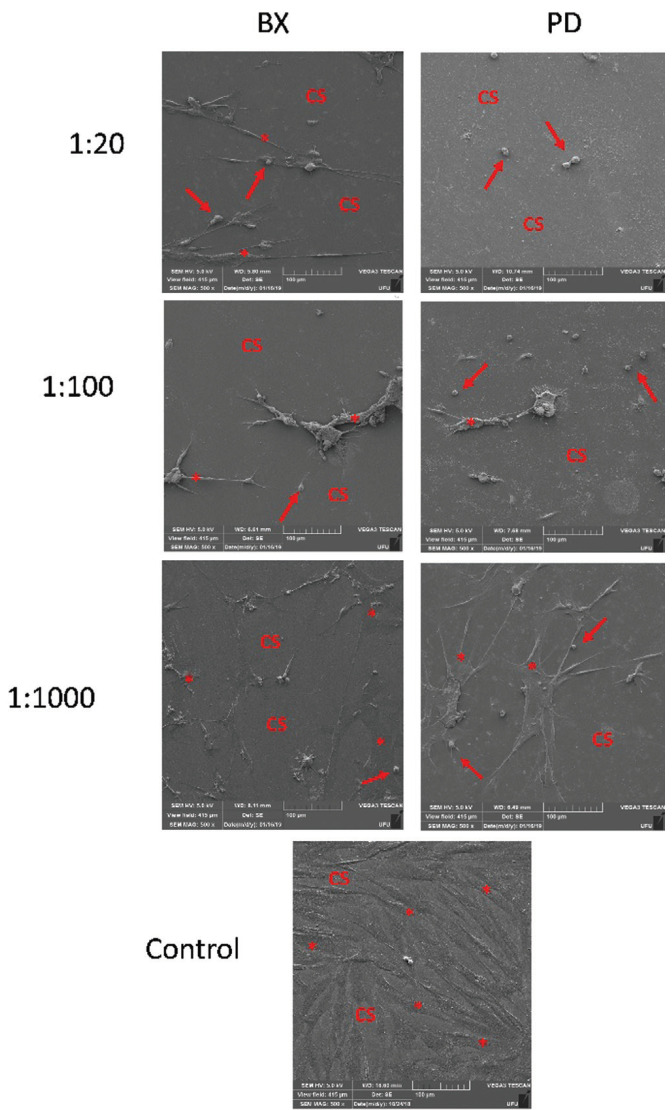
Images representative of pulp cells obtained by scanning electron microscopy after application of the different materials (Brix 3000 - BX and Papacarie Duo - PD) and their concentrations (1:20, 1:100, and 1:1000) in comparison with those in the control group. CS indicate the coverslip surface, stars indicate the cytoplasmic prolongation of pulp cells and arrows indicate contracted pulp cells. 500x magnification, 5.0 kV.

The SEM images of the control group showed the normal appearance of primary pulp culture, with a large number of fusiform cells (elongated shape) and cytoplasmic filaments covering the coverslips. In the 1:1000 dilution for both materials, the images indicated a reduction in the number of cells and the cytoplasmic extensions, in addition to a cellular contraction. These changes were even more evident in the highest concentrations of both Brix and Papacárie, with an exacerbated decrease in the number of cells and an increase in these morphological changes (Fig. 1).

-Elementary analysis by energy-dispersive X-ray spectroscopy (EDS)

It could be observed that for the Ca and *P* elements in isolation and the Ca/P ratio, there was no difference between the control group and both chemomechanical caries removal agents tested (*p*>0.05). The group treated with acid presented the lowest values of Ca, P, and Ca/P, differing statistically significantly from the other groups (*p*<0.05) (Table 3).

**Table 3:**
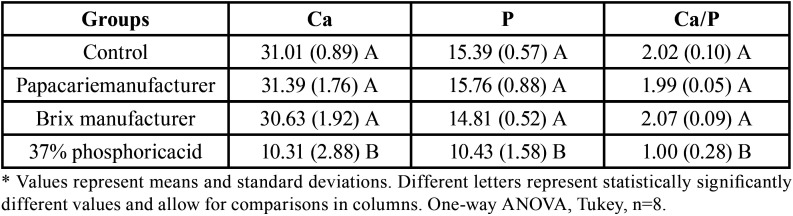
Rates of the specific elements (calcium, phosphorus, and Ca/P ratio) found in the dentinal discs through EDS analysis, at the minimum time recommended by the manufacturer.

-Morphology of dentinal discs by Scanning Electron Microscopy (SEM)

**Figure 2:**
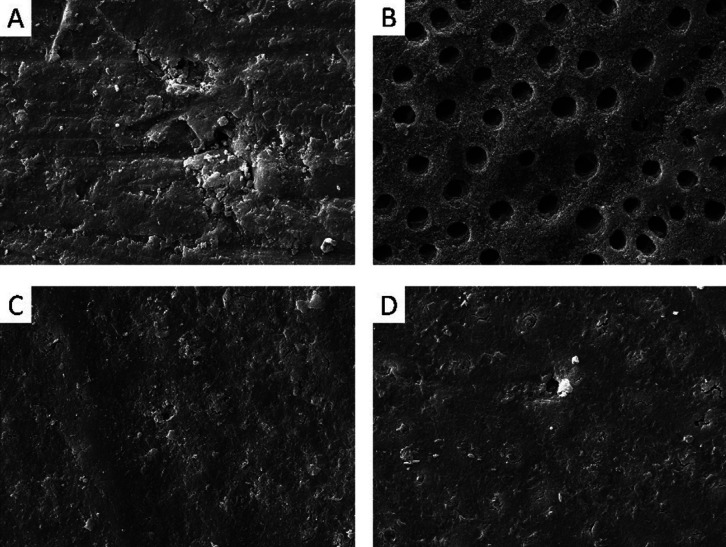
Morphological aspects of the dentinal tubules in the dentin discs obtained by scanning electron microscopy after application of the different materials: the negative control group (without material application - A), phosphoric acid (B), Brix 3000 (C) andPapacarie Duo (D) at the minimum times indicated by the manufacturers. 5000x magnification, 5.0 KV.

The images obtained for the discs showed a difference in the removal of the smear layer of the Brix 3000 and Papacarie Duo materials compared with the positive control (phosphoric acid), but there was no difference from the negative control (without material application). That is, the phosphoric acid was able to remove a significant portion of the smear layer in the dentinal tubules, while the Brix 3000 and Papacarie Duo materials did not present such ability, showing the tubules to be completely or partially obliterated (Fig. 2).

## Discussion

The present study assessed the cytotoxicity of two chemomechanical caries removal agents (Brix 3000™ and Papacarie Duo™) when applied directly to the pulp cells of deciduous teeth at different dilutions, as well as the morphology and chemical composition of the dentin tissue after the application of these materials. It has been confirmed that the chemomechanical removal of caries is considered one of the most conservative and effective methods in certain treatments (21), and extensive research has been performed regarding its clinical efficacy, decrease in patient discomfort, and the long-term success of the procedure (6). However, the presence of studies assessing the effects of chemomechanical caries removal agents on the dentin-pulp complex, especially in deciduous teeth, is still scarce in the literature. Thus, for the safe clinical use of these materials, previous assessments regarding their efficacy in tissue removal and information on their potential effects on pulp tissue become relevant. As for the cytotoxicity of the materials, the null hypothesis was rejected, since both materials presented cytotoxicity in direct contact. Regarding the dentinal composition after the application of the materials, the null hypothesis was accepted, because the dentinal structure was not changed by any of the materials tested.

The results of the cytotoxicity tests (MTT and TB) showed that both materials reduced the metabolism and the number of viable cells compared with the control group and that the lower the dilution, the higher the cytotoxicity. Analysis of these data agrees with the study performed by Garcia-Contreras *et al*. (22), who tested two chemomechanical caries removal agents (Papacarie Duo and Carisolv) in normal and cancerous oral cells. Moreover, it is worth noting that the dilutions used were similar to those assessed in the present study. Papacarie Duo significantly reduced the number of viable cells, especially at the lowest dilutions. However, even at high dilutions, Papacarie Duo showed cytotoxicity, differing from Carisolv, which presented low or no reduction of viable cells. In the present study, the Brix 3000 material presented lower cytotoxicity than Papacarie Duo at all dilutions, which also agrees with a recent study that performed the MTT assay for the same materials (BX and PD) (17).

Clinically, these data are important because it is known that the cytotoxicity of the material may lead to postoperative sensitivity and pulp necrosis. It is worth noting that dentin permeability and thickness are relevant factors at the moment of application (23). However, the dentist should use the material with caution to prevent damage to the dentin-pulp complex. Further *in vitro* studies are still required, assessing the transdentinal action of these materials, as well as clinical studies to identify more precisely the amount of material to be used per session, time of application, and whether the indication should relate to cavity depth. The minimum time of action of the materials for clinical practice is a limiting factor for BX, in which 2 min can make patient management difficult, especially with pediatric patients, necessitating studies evaluating its effectiveness in less time.Additionally, regarding cytotoxicity in the direct contact of materials, another study showed that chemomechanical caries removal agents such as Papacarie™ may present cytotoxicity for pulp cells (20). The authors observed that the Papacarie™ material presented cytotoxicity only 50s after application, while after 24 h it was not cytotoxic. It was suggested that the gel presents cytotoxicity only initially, when it is more active, with no ability to damage the pulp tissue after 24 h.

Regarding the indirect contact of chemomechanical caries removal agents with the pulp tissue, the current literature contains no transdentinal studies with these materials that provide data for comparison. Another observation is that several materials commonly used in clinical practice are not considered cytotoxic for the pulp when analyzed indirectly, as in a study assessing transdentinal cytotoxicity of three different resin cements on pulp cells. The results showed a decrease in viable cells but were not sufficient to characterize the material as cytotoxic (24). According to the safety recommendations of the ISO 10993-5: 1999 (E), to be considered cytotoxic, there must be a reduction of at least 30% of viable cells. Transdentinal studies with materials used for pulp capping have shown that there is also no decrease in cell viability, as seen in the study by Cavalcanti *et al*. (25), concluding that the materials would not be able to release cytotoxic substances to the pulp tissue. The cytotoxicity assessment in bleaching agents is also performed often, because these materials present high diffusion in the dental tissue. A recent study showed cytotoxicity for some bleaching agents even with the dentin barrier of 2 mm, but there was no correlation between the amount of peroxide disseminated and the product concentration (26). That is, a certain bleaching gel assessed, even at concentrations equal to others, presented higher diffusion in the dentinal tissue and therefore higher cytotoxicity for pulp cells. In the same study, the gel that presented a higher decrease in cell viability when tested *in vivo* (rats) presented severe necrosis of the crown and root pulp (26).

For analysis of the morphological aspects of pulp cells obtained by SEM, there were no differences between both materials, but the morphological difference of the cells compared with those of the control group was evident. After contact with both materials, there was a decrease in the number of cells and changes in the morphology, confirming the presence of cell stress. The SEM, in this case, is complementary to the cell cytotoxicity tests. Despite the lack in the literature of similar studies performed with chemomechanical caries removal agents, it is of utmost importance that these tests be performed in combination because they make the results related to cytotoxicity more real and representative. At morphological evaluating dentinal discs, phosphoric acid was used as a positive control because it is known that acid-etching promotes significant smear layer removal from the dentinal tubules and dentinal demineralization (27). The present study assessed whether the smear layer removal would be similar to that in the positive control. The images produced when the cells were exposed to the materials, following the minimum time recommended by the manufacturer, showed no differences between the chemomechanical caries removal agents and the negative control group (absence of material). That is, the removers were not able to unblock the dentinal tubules. A strong similarity was observed between the dentin treated with Papacarie Duo and Brix 3000 and the untreated dentin, showing that the materials tested did not interfere significantly with the tubular dentin surface.

The assessments of Ca and P, as well as the Ca/P ratio, allow for the establishment of a reliable pattern of behavior of the chemical elements in the dentin, regardless of the variation of the other elements (28). The values of the ratio found in the present study agree with those reported in other studies (29). As for the ratio of Ca/P to acid, a decrease was observed compared with that in healthy dentin, which is probably due to the composition of the material itself. This has also been evidenced in a recent study, but the methodology was not similar to that used in this study (27).It was observed that the levels of calcium and phosphorus, for both materials, did not differ statistically significantly from those of the control group (untreated dentin). This shows that current chemomechanical caries removal agents cannot change the dentin structure, which agrees with studies reported in the literature performed with other chemomechanical caries removal agents (8).

In terms of multiple methodologies, the EDS presents some limitations. First, false-positive results may occur from the high penetration of energy, but precision may reach 91-95%. Moreover, the radiation absorbed is only around 1%, but increasing this dose may result in damage to the sample (30).Additionally, because this was a laboratory study, the data presented cannot be extrapolated directly to the dental clinic, and further laboratory and clinical studies are required aimed at the production of a usage protocol for chemomechanical caries removal agents able to remove carious tissue effectively without damaging the pulp tissue, considering the principles of minimally invasive caries excavation techniques.

## Conclusions

The Papacarie Duo and Brix 3000 chemomechanical caries removal agents showed cytotoxicity in direct contact with the pulp cells from deciduous teeth. The Brix 3000 material presented less cytotoxicity than Papacarie Duo. Both materials did not significantly change the dentinal structure.

## References

[1] Richards D (2013). Oral diseases affect some 3.9 billion people. Evid Based Dent.

[2] Fusayama T (1979). Two layers of carious dentin; diagnosis and treatment. Oper Dent.

[3] Bjørndal L, Fransson H, Bruun G, Markvart M, Kjældgaard M, Näsman P (2017). Randomized clinical trials on deep carious lesions: 5-year follow-up. J Dent Res.

[4] Ngo HC, Mount G, McIntyre J, Tuisuva J, Von Doussa RJ (2006). Chemical exchange between glass-ionomer restorations and residual carious dentine in permanent molars: an in vivo study. J Dent.

[5] Maltz M, de Oliveira EF, Fontanella V, Bianchi R (2002). A clinical, microbiologic, and radiographic study of deep caries lesions after incomplete caries removal. Quintessence Int.

[6] Bittencourt ST, Pereira JR, Rosa AW, Oliveira KS, Ghizoni JS, Oliveira MT (2010). Mineral content removal after Papacarie application in primary teeth: a quantitative analysis. J Clin Pediatr Dent.

[7] Al-Halabi M, Salami A, Alnuaimi E, Kowash M, Hussein I (2020). Assessment of paediatric dental guidelines and caries management alternatives in the post COVID-19 period. A critical review and clinical recommendations. Eur Arch Paediatr Dent.

[8] Decup F, Lasfargues JJ (2014). Minimal intervention dentistry II: part 4. Minimal intervention techniques of preparation and adhesive restorations. The contribution of the sono-abrasive techniques. Br Dent J.

[9] Jew J, Chan KH, Darling CL, Fried D (2017). Selective removal of natural caries lesions from dentin and tooth occlusal surfaces using a diode-pumped Er:YAG laser. Proc SPIE Int Soc Opt Eng.

[10] Safwat O, Elkateb M, Dowidar K, Salam HA, El Meligy O (2018). Microbiological evaluation of ozone on dentinal lesions in young permanent molars using the stepwise excavation. J Clin Pediatr Dent.

[11] Deng Y, Feng G, Hu B, Kuang Y, Song J (2018). Effects of Papacarie on children with dental caries in primary teeth: a systematic review and meta-analysis. Int J Paediatr Dent.

[12] Abdul Khalek AMG, Elkateb MA, Abdel Aziz WE, El Tantawi M (2017). Effect of Papacarie and alternative restorative treatment on pain reaction during caries removal among children: a randomized controlled clinical trial. J Clin Pediatr Dent.

[13] Habib CM, Kronman J, Goldman MA (1975). A chemical evaluation of collagen and hydroxyproline alter treatment with GK-101(N-chloroglycine). PharmacolTher Dent.

[14] Cecchin D, Farina AP, Orlando F, Brusco EH, Carlini B (2010). Effect of Carisolv and Papacarie on the resin-dentin bond strength in sound and caries-affected primary molars. Braz J Oral Sci.

[15] Motta LJ, Martins MD, Porta KP, Bussadori SK (2009). Aesthetic restoration of deciduous anterior teeth after removal of carious tissue with Papacárie. Indian J Dent Res.

[16] Bussadori SK, Guedes CC, Bachiega JC, Santis TO, Motta LJ (2011). Clinical and radiographic study of chemical-mechanical removal of caries using Papacárie: 24-month follow up. J Clin Pediatr Dent.

[17] Santos TML, Bresciani E, Matos FS, Camargo SEA, Hidalgo APT, Rivera LML (2020). Comparison between conventional and chemomechanical approaches for the removal of carious dentin: an in vitro study. Sci Rep.

[18] Krithikadatta J, Gopikrishna V, Datta M (2014). CRIS guidelines (checklist for reporting in-vitro studies): a concept note on the need for standardized guidelines for improving quality and transparency in reporting in-vitro studies in experimental dental research. J Conserv Dent.

[19] Gronthos S, Mankani M, Brahim J, Robey PG, Shi S (2000). Postnatal human dental pulp stem cells (DPSCs) in vitro and in vivo. ProcNatlAcadSci USA.

[20] Miyagi SPH, Mello I, Bussadori SK, Marques MM (2006). Resposta de fibroblastos pulpares humanos em cultura ao gel de papacárie® (Response of cultured pulpal fibroblasts to papacárie® gel). Revista de Odontologia da Universidade Cidade de São Paulo.

[21] Bohari MR, Chunawalla YK, Ahmed BM (2012). Clinical evaluation of caries removal in primary teeth using conventional, chemomechanical and laser technique: an in vivo study. J Contemp Dent Pract.

[22] Garcia-Contreras R, Scougall-Vilchis RJ, Contreras-Bulnes R, Kanda Y, Nakajima H, Sakagami H (2014). Cytotoxicity and pro-inflammatory action of chemomechanical caries-removal agents against oral cells. In Vivo.

[23] Costa CA, Ribeiro AP, Giro EM, Randall RC, Hebling J (2011). Pulp response after application of two resin modified glass ionomer cements (RMGICs) in deep cavities of prepared human teeth. Dent Mater.

[24] Garcia LFR, Pontes EC, Basso FG, Hebling J, de Souza Costa CA, Soares DG (2016). Transdentinal cytotoxicity of resin-based luting cements to pulp cells. Clin Oral Investig.

[25] Cavalcanti BN, Rode SM, Marques MM (2005). Cytotoxicity of substances leached or dissolved from pulp capping materials. IntEndod J.

[26] Llena C, Collado-González M, García-Bernal D, Oñate-Sánchez RE, Martínez CM, Moraleda JM (2019). Comparison of diffusion, cytotoxicity and tissue inflammatory reactions of four commercial bleaching products against human dental pulp stem cells. Sci Rep.

[27] de Los Angeles Moyaho-Bernal M, Contreras-Bulnes R, Rodríguez-Vilchis LE, Rubio-Rosas E, Scougall-Vilchis RJ, Centeno-Pedraza C (2018). Morphological and chemical changes in human deciduous dentin after phosphoric acid, self-etching adhesive and Er: YAG laser conditioning. Microsc Res Tech.

[28] Zamudio-Ortega CM, Contreras-Bulnes R, Scougall-Vilchis RJ, Morales-Luckie RA, Olea-Mejía OF, Rodríguez-Vilchis LE (2014). Morphological and chemical changes of deciduous enamel produced by Er:YAG laser, fluoride, and combined treatment. Photomed Laser Surg.

[29] Arnold WH, Gaengler P (2007). Quantitative analysis of the calcium and phosphorus content of developing and permanent human teeth. Ann Anat.

[30] Hall TA, Gupta BL (1984). The application of EDXS to the biological sciences. J Microsc.

